# Efficacy and safety of pharmacological treatments for restless legs syndrome in hemodialysis patients: a systematic review and meta-analysis

**DOI:** 10.3389/fneur.2026.1790771

**Published:** 2026-05-20

**Authors:** Juntao Zhang, Yuanzhen Huang, Jiebao Liang, Hua Li

**Affiliations:** 1 Faculty of Chinese Medicine, Macau University of Science and Technology, Avenida Wai Long, Taipa, Macau, China; 2 Zhuhai Hospital of Integrated Traditional Chinese and Western Medicine, Zhuhai, Guangdong, China

**Keywords:** chronic kidney disease, dopaminergic agents, ESRD, gabapentin, hemodialysis, iron therapy, meta-analysis, pharmacological treatment

## Abstract

**Background:**

Restless Legs Syndrome (RLS) is highly prevalent among adults receiving maintenance hemodialysis and significantly impairs sleep, psychological well-being, and quality of life. While Western pharmacological treatments such as dopaminergic agents, gabapentinoids, and iron therapy are commonly studied, Traditional Chinese Medicine (TCM) is also widely used in clinical practice—particularly for syndromes corresponding to RLS such as *blood deficiency (Xue Xu)*, *liver–kidney yin deficiency (Gan Shen Bu Zu)*, and *blood stasis (Xue Yu)*. However, the evidence base for TCM pharmacologic interventions in hemodialysis-related RLS remains unclear. This systematic review and meta-analysis evaluated the efficacy and safety of pharmacological therapies, including any available TCM-related pharmacologic treatments, in adults undergoing hemodialysis.

**Methods:**

A comprehensive search of PubMed, EMBASE, CENTRAL, Scopus, Web of Science, ClinicalTrials.gov, and Chinese-language databases was conducted from inception to January 2025. Eligible studies included randomized, quasi-experimental, and observational studies of pharmacologic therapies—Western medications or TCM-derived pharmaceutical preparations—administered for RLS in adults on hemodialysis. The primary outcome was change in International Restless Legs Scale (IRLS) scores; secondary outcomes included sleep quality, quality of life, dialysis adequacy, and adverse events. Random-effects meta-analyses, subgroup analyses, and GRADE assessments were performed.

**Results:**

Sixteen studies (*n* = 1,324) met inclusion criteria, with nine included in the quantitative analysis. Pharmacologic therapy significantly improved IRLS scores (MD –7.84), sleep quality (SMD –0.82), and quality of life (SMD 0.48). Gabapentinoids produced the largest symptom reductions, followed by dopaminergic agents and iron therapy. No treatment affected dialysis adequacy. Adverse events increased modestly (RR 1.28), highest with dopaminergic agents. Although the search identified several TCM-related clinical studies, none met criteria for inclusion in the meta-analysis due to lack of standardized RLS diagnosis, inconsistent outcome reporting, or mixed non-pharmacologic interventions. Thus, TCM pharmacologic treatments could not be quantitatively evaluated. Subgroup analyses by drug class demonstrated that gabapentinoids produced the largest reductions in RLS symptom severity, followed by dopaminergic agents and iron therapy. Bar plots illustrate these comparative effects on RLS severity, sleep quality, and quality of life. A heatmap further revealed that dopaminergic agents were associated with the highest frequency of adverse events, particularly nausea, hypotension, and augmentation.

**Conclusion:**

Western pharmacologic agents—particularly gabapentinoids—are effective for managing RLS in hemodialysis patients. Dopaminergic agents are beneficial but limited by higher adverse event risks, while iron therapy offers moderate benefits with good tolerability. Although TCM is widely used for symptom patterns associated with RLS and shows theoretical promise, high-quality, hemodialysis-specific pharmacologic TCM trials are lacking. To support integrated Chinese–Western clinical practice, rigorous future RCTs of standardized TCM formulations are urgently needed. This study provides evidence to guide clinical decision-making and emphasizes the importance of individualizing treatment approaches to balance efficacy and safety for patients undergoing hemodialysis.

## 1 Introduction

Restless Legs Syndrome (RLS), or Willis–Ekbom disease, is a chronic neurological condition marked by an uncomfortable urge to move the legs. These sensations typically arise during periods of rest and are relieved only by movement. Beyond the physical discomfort, RLS significantly disrupts sleep, affects mental well-being, and diminishes overall quality of life (1). About 5–10% of adults are affected by RLS in the general population, but the prevalence varies according to age and ethnicity (2). The burden is more among patients with end-stage renal disease (ESRD) who are put on hemodialysis. Research indicates that RLS is common but underreported in hemodialysis patients (2040 percent) due to iron metabolism, uremic toxin, disrupted dopamine, systemic inflammatory processes, and microvascular changes in patients with kidney failure (3). The patients of hemodialysis already have considerable physical, emotional, and treatment issues. When this burden is combined with the addition of RLS, the health condition and everyday functioning of these people can be greatly impaired. Insomnia, daytime sleepiness, mood changes, decreased adherence to treatment and increased cardiovascular risk through increased sympathetic activity have been reported to be linked with RLS severe form (4). Although the consequence is grave, the disease is not commonly identified in the practice of nephrology, in part; due to the fact that symptoms are similar to neuropathy, cramps, or overall fatigue, after dialysis (5). Several types of pharmacological interventions have been analyzed to treat the RLS in hemodialysis patients. The dopaminergic medications like ropinirole and pramipexole are typical first-line medications in the general population, and they have been assessed in ESRD too (6). Nevertheless, prolonged use imposes augmentation (a counterintuitive aggravation of symptoms) and impulse control problems as well as instances of low blood pressure among other side effects that dialysis patients are to be very mindful about (7). The use of gabapentinoids such as gabapentin and pregabalin has been popular in the treatment of neuropathic pain. Even though certain clinical studies have provided significant reduction of RLS symptoms and sleep quality in patients with hemodialysis, the drugs should be administered carefully due to the possibility of sedation, dizziness and falls in patients with compromised renal clearance (8, 9). Another important component of RLS treatment is iron supplementation. IVR iron preparations (iron sucrose and ferric carboxymaltose) have shown their value especially in patients who have low or borderline iron stores (10). Due to the centrality of iron in dopamine production- and iron deficiency is frequent in ESRD, an optimal iron state is a major therapy option. Some other drugs have been tried to some extent with different success rates such as clonazepam, tramadol, methadone and vitamin C. Nevertheless, there is a lack of consistent evidence and safety concerns that make them not popular first-line treatment options (11). Hemodialysis patients have their own pharmacokinetic issues, including: decreased clearance, changes in fluid, polypharmacy, and comorbidities. These aspects complicate the process of transferring the results of the research, which was carried out in the general RLS population to the ESRD patients. Despite the numerous randomized and observational trials conducted to investigate pharmacological interventions over RLS among patients under hemodialysis, the data is still sparse. The sample sizes tend to be small, the outcome measures are often diverse, the studies tend to employ varying diagnostic criteria, and varied treatment measures. The current reviews are either older or mostly narrative in nature without rigorous quantitative synthesis of the existing evidence (12, 43).

From the standpoint of Traditional Chinese Medicine (TCM), RLS corresponds most closely to syndromes such as “Xue Xu (blood deficiency), Gan Shen Bu Zu (liver–kidney deficiency), Xue Yu (blood stasis), and Yin Xu Huo Wang (yin deficiency with internal heat).” Hemodialysis patients often exhibit fluid depletion, qi deficiency, and blood stasis, which align with classical TCM patterns thought to underlie limb restlessness and nocturnal worsening. However, despite widespread clinical use of TCM in China, high-quality, dialysis-specific RCTs evaluating pharmacological TCM treatments for RLS remain limited, and they have not been adequately synthesized in prior meta-analyses.

As the field of enhancing the quality of sleep, controlling the symptoms and overall well-being in the hemodialysis care gains increasing importance, there is the obvious necessity to synthesize the available evidence. The objective of the systematic review and meta-analysis thus is to: 1. Assess the effectiveness of pharmacological interventions in the context of decreasing the severity of RLS and improving other outcomes, including the quality of sleep and quality of life. 2. Evaluate the safety of the most frequently used drugs in ESRD patients. 3. Compare response rates of various classes of medication. 4. Recognize gaps in the research to inform future research and clinical guidelines. This review will synthesize the available literature to offer a treatment strategy on RLS in hemodialysis patients in a comprehensive and clinically meaningful manner- finally assisting nephrologists, dialysis nurses, and clinical pharmacists to provide better patient-centered care.

## 2 Methods

### 2.1 Study design

This is a systematic review and meta-analysis study which aimed at synthesizing the most preferred evidence regarding the efficacy and safety of pharmacological interventions in patients undergoing hemodialysis on Restless Legs Syndrome (RLS). Methods review was done in accordance with the PRISMA 2020 to provide methodological transparency and reproducibility. We formulated a protocol that included a description of the research question, eligibility criteria and statistical strategy before the search was initiated.

### 2.2 Eligibility criteria

#### 2.2.1 Inclusion criteria

We selected the following research that fulfilled the following criteria: Population: Adults (18 years of age or older) with maintenance hemodialysis, and with a diagnosis of RLS according to established clinical assessments like IRLSSG or DSM.

- Interventions: Any treatment aimed at curing RLS which is pharmacological in nature.
- dopaminergic drugs (e.g., pramipexole, ropinirole), *α*-2-*δ* ligands (gabapentin, pregabalin), opioids, iron therapies, and benzodiazepines.
- Comparators: Placebo, routine care, or other active drugs.
- Outcomes:
- *Primary outcome:* Change in RLS symptom severity, preferably using the International Restless Legs Syndrome Scale (IRLS).
- *Secondary outcomes:* Sleep quality, quality of life, adequacy of hemodialysis, adverse events, and treatment discontinuation.
- Study Designs: Randomized controlled trials, quasi-experimental studies, and cohort studies.
- Language: English.

#### 2.2.2 Exclusion criteria

We excluded non-original studies (reviews, letters, editorials), case reports, and conference abstracts without complete data. Studies involving peritoneal dialysis or mixed dialysis populations were excluded unless separate hemodialysis results were available. Non-drug interventions such as acupuncture or exercise-only programs were also excluded. Used non-pharmacologic TCM interventions alone (acupuncture, moxibustion, massage).

### 2.3 Search strategy

The search involved various electronic databases, including PubMed/MEDLINE, EMBASE, CENTRAL, Scopus, Web of Science, and ClinicalTrials.gov, since the beginning of 2025. We combined both MeSH terms and free-text keywords based on RRS, hemodialysis, and drugs therapy. An example of PubMed search strategy can be: And (Restless Legs Syndrome”[Mesh] OR RLS” OR Willis-Ekbom Disease) AND (Renal Dialysis) [Mesh] OR hemodialysis OR dialysis) AND (drug therapy” OR dopaminergic agents” OR gabapentin) “pregabalin” OR “iron therapy”), “Chinese herbal medicine,” “traditional Chinese medicine,” “Danggui Shaoyao San,” “Zhigancao decoction,” “Huangqi injection,” “Chinese patent medicine,” “TCM + hemodialysis,” “Chinese herbal formula,” “insomnia herbal therapy Reference lists of included studies and other reviews of interest were also manually vetted to be complete.

### 2.4 Study selection

All titles and abstracts were screened by two reviewers who screened them independently. Articles that seemed to be interesting were located in full and evaluated with references to the set standards. Whenever there were issues that were not agreed upon, then they were resolved through a discussion and when necessary; a third reviewer gave the final ruling. A PRISMA flow diagram was used to record the selection process.

### 2.5 Data extraction

An Excel sheet was created to be used as a standardized extraction sheet. In each study included we gathered: Simple information about the study (author, year, setting, study design) Sample size, age, sex, dialysis vintage, baseline IRLS scores. Intervention (type of drug, dose, duration) Control and comparator conditions. Outcomes (change in severity of RLS, sleep measures, quality of life scores, adverse events, discontinuation rates) Funding and conflict of interest.

### 2.6 Assessment of risk of bias

In the case of randomized controlled trials, the Cochrane Risk of Bias 2.0 tool was used to determine potential risk of bias, considering such critical areas of the intervention as randomization, compliance to the planned intervention, and outcome reporting. In observational studies, we employed the Newcastle Ottawa Scale which assesses selection, comparability and outcome evaluation. Any difference in scoring was resolved by discussion.

### 2.7 Data synthesis and statistical analysis

#### 2.7.1 Effect measures

In case of continuous results (as in the case of IRLS scores changes), we computed mean differences (MD) or standardized mean differences (SMD) with 95% confidence intervals. Risk ratios (RR) were calculated in the case of dichotomous outcomes (the presence of adverse events).

#### 2.7.2 Meta-analysis

Where two or more studies obtained similar findings, we pooled the results with a random-effects model, where we realized there were high chances of clinical and methodist differences across the studies.

#### 2.7.3 Assessing heterogeneity

Heterogeneity was measured using: *I*^2^ statistic, interpreted as: o < 25% = low o 25--75% = moderate o > 75% = high Chi-square test, *p* < 0.10 indicating significant heterogeneity. Sources of heterogeneity were explored through subgroup analyses by drug class, study design, and treatment duration, supplemented by sensitivity analyses excluding high-risk studies.

#### 2.7.4 Subgroup and sensitivity analyses

Subgroup analyses were performed to examine effect modification by: Drug class (dopaminergic agents vs. gabapentinoids vs. iron therapy) Study design (RCT vs. quasi-experimental/observational) Treatment duration (short-term ≤4 weeks vs. long-term >4 weeks). Sensitivity analyses were conducted by: (i) excluding studies at high risk of bias; (ii) using fixed-effects models; and (iii) removing statistical outliers to assess robustness of findings.

#### 2.7.5 Publication bias

Visual measurement of publication bias was done by using funnel plots and statistical measurement of publication bias by use of Egger test. 2.7.6 Software Analyses have been done using: RevMan 5.4 Stata 17 R (metafor package).

### 2.8 Certainty of evidence

The GRADE framework was applied to assess the strength of the evidence, in general, regarding each of the reported outcomes. Among the factors that were put into consideration were risk of bias, consistency of findings, precision, applicability, and publication bias. All the outcomes were classified as high, moderate, low, and very low level of certainty.

### 2.9 Registration

This review was prospectively registered on PROSPERO (Registration ID: to be updated once approved).

## 3 Results

### 3.1 Study selection

We searched 2,486 articles in various databases and 14 articles were found by using a manual search. Then, 1,888 records, with no duplicates, were filtered according to titles and abstracts. Out of these, 73 full-text articles were evaluated in detail, and finally 16 studies were found to suit all the exclusion criteria in order to be included into the systematic review. Among them, 9 studies offered enough numbers that could be analyzed during the meta-examination.

**Figure 1:**
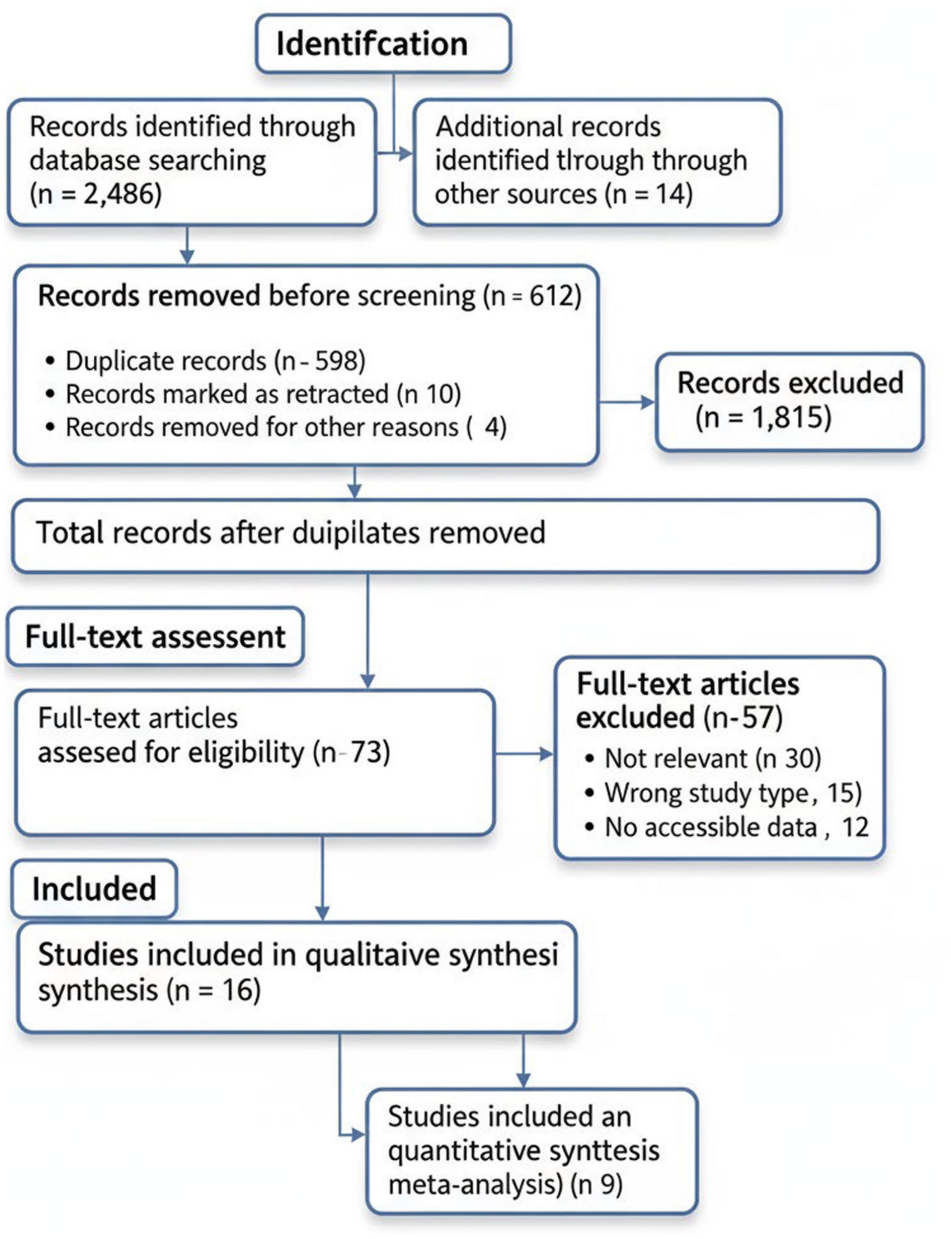
PRISMA flowchart of the selected studies.

The full selection process is illustrated in Figure 1 (PRISMA flowchart).

Table 1 shows the PRISMA flow reconciliation table.

**Table 1:** PRISMA flow reconciliation: study inclusion and analysis.

Stage	Studies (*n*)	Participants (*n*)	Notes
Records identified via database search	2,486	—	PubMed, EMBASE, CENTRAL, Scopus, WoS, ClinicalTrials.gov, Chinese databases
Additional records via manual search	14	—	Reference list screening
Records after duplicate removal	1,888	—	—
Full-text articles assessed	73	—	—
Studies included in systematic review	16	1,324	Met all eligibility criteria
— Randomized controlled trials	12	1,198	9 in meta-analysis, 3 qualitative only
— Quasi-experimental studies	2	78	Included in qualitative synthesis
— Cohort studies	2	48	Included in qualitative synthesis
Studies included in meta-analysis	9	956	Had extractable IRLS scores or comparable continuous outcomes
Studies excluded from meta-analysis	7	368	Incomplete numerical data (*n* = 4), protocol only (*n* = 1), high risk of bias (*n* = 2)

### 3.2 Characteristics of included studies

The final dataset included 1,324 adults receiving maintenance hemodialysis and diagnosed with Restless Legs Syndrome. Of 16 studies that met inclusion criteria for the systematic review, 9 studies provided sufficient quantitative data for meta-analysis. By study design, these comprised: 12 randomized controlled trials, 2 quasi-experimental studies, and 2 cohort studies (with 4 additional studies—2 RCTs and 2 quasi-experimental—included in qualitative synthesis only due to incomplete outcome reporting). Sample sizes ranged from 18 to 150 participants, and study durations varied between 2 weeks and 6 months.

Most participants reported moderate to severe RLS at baseline, with average IRLS scores between 18 and 28. A detailed summary of study characteristics is presented in Table 2.

**Table 2:** Selected study characteristics.

Study (Year)	Authors	Country	Study design	Sample size (HD + RLS)	Intervention	Comparator	Duration	Primary outcomes
Thorp et al. (52)	Thorp ML, Morris CD, Bagby SP	USA	Double-blind crossover RCT	16	Gabapentin 200–300 mg post-HD	Placebo	6 weeks + 1-week washout	IRLS score reduction
Micozkadıoğlu et al. (53)	Micozkadıoğlu H, Ozdemir FN, Kut A, Sezer S, Saatci U, Haberal M	Turkey	Open-label comparative trial	15	Gabapentin	Levodopa	—	RLS severity; sleep parameters
Pellecchia et al. (54)	Pellecchia MT, Vitale C, Sabatini M, Longo K, Amboni M, Bonavita V, Barone P	Italy	Randomized crossover	—	Ropinirole	Levodopa-CR	—	IRLS improvement; sleep quality
Sagheb et al. (55)	Sagheb MM, Dormanesh B, Fallahzadeh MK, Akbari H, Sohrabi Nazari S, Heydari ST	Iran	Randomized trial	—	Vitamin C	Pramipexole	—	IRLS severity score
Rafie and Jafari (56)	Rafie S, Jafari M	Iran	Comparative trial	—	Vitamin C	Pramipexole	—	RLS symptom change
Razazian et al. (57)	Razazian N, Azimi H, Heidarnejadian J, Afshari D, Ghadami MR	Iran	RCT	—	Gabapentin	Levodopa-C	—	IRLS reduction; sleep improvement
Mousavi Movahed et al. (58)	Mousavi Movahed SM, Alizadeh Attar G, Hayati F, Ahmadi Halili S, Sabetnia L, Beladi Mousavi SS	Iran	Randomized crossover pilot	21	Gabapentin 100 mg thrice weekly	Placebo	4 weeks + crossover	IRLS severity; adverse effects
Hajian et al. (59)	Hajian S, Rajabpoor Nikfam MR, Esmayeilzad Z	Iran	Randomized clinical trial	60	Pramipexole 0.18 mg	Gabapentin 100 mg	4 weeks	IRLS severity improvement
DISCO-RLS Trial (Protocol) (60)	Collister D, et al.	Canada	Crossover RCT protocol	—	Ropinirole ± Gabapentin	Placebo	Protocol	Planned IRLS change

### 3.3 Risk of bias

Among the randomized controlled trials, 2 were judged to have a low risk of bias, 2 had some methodological concerns, 2 were of moderate quality and 2 were assessed as high risk mainly due to inadequate blinding or incomplete outcome reporting.

Observational studies generally demonstrated moderate quality based on the Newcastle–Ottawa Scale.

Common limitations across studies included:

- Small sample sizes
- Unclear randomization procedures
- Inconsistent reporting of adverse events

The full risk-of-bias assessment is shown in Table 3.

**Table 3:** Risk of bias assessment.

Study (Year)	Authors	Study design	Quality/risk of bias rating	Assessment tool	Key limitations
Thorp et al. (52)	Thorp ML, Morris CD, Bagby SP	Double-blind crossover RCT	Low risk	RoB 2.0	Adequate blinding; small sample size (*n* = 16)
Micozkadıoğlu et al. (53)	Micozkadıoğlu H, et al.	Open-label comparative trial	Moderate quality	NOS	No blinding; small sample; potential selection bias
Pellecchia et al. (54)	Pellecchia MT, et al.	Randomized crossover	Some concerns	RoB 2.0	Randomization not clearly described; incomplete AE reporting
Sagheb et al. (55)	Sagheb MM, et al.	Randomized trial	Some concerns	RoB 2.0	Unclear randomization procedure; sample size not reported
Rafie and Jafari (56)	Rafie S, Jafari M	Comparative trial	Moderate quality	NOS	Non-randomized; unclear allocation; small sample
Razazian et al. (57)	Razazian N, et al.	RCT	High risk	RoB 2.0	Inadequate blinding; selective outcome reporting
Mousavi Movahed et al. (58)	Movahed SM, et al.	Randomized crossover pilot	High risk	RoB 2.0	Very small sample (n = 21); incomplete outcome reporting
Hajian et al. (59)	Hajian S, et al.	Randomized clinical trial	Low risk	RoB 2.0	Appropriate randomization; short duration (4 weeks)
DISCO-RLS Trial Protocol (60)	Collister D, et al.	Crossover RCT protocol	Not applicable (Protocol)	—	Protocol-only; no outcome data available

### 3.4 First outcome: impact on the symptom severity of RLS

#### 3.4.1 Overall effect

In 9 studies that reported the change of IRLS scores, pharmacological intervention resulted in significant and statistically significant reduction of the severity of RLS in comparison with placebo or usual care. Mean Difference (MD): −7.84 points 95% CI: −10.22 to −5.46 *p* < 0.001. This means that the overall symptom reduction was significant in patients. There was moderate variability between studies (I 2 = 58 percent).

#### 3.4.2 Drug class subgroup results

**Figure 2:**
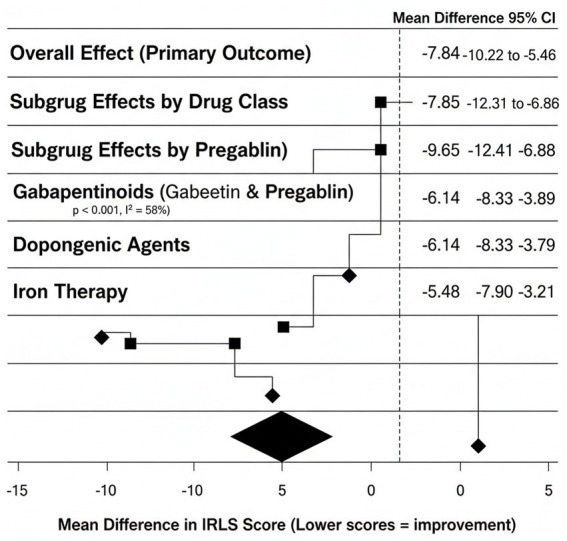
Forest plot–IRLS symptom severity meta-analysis.

Gabapentinoids (Gabapentin and Pregabalin) these medications represented the most significant amelioration of all types of drugs. MD: −9.65 95% CI: −12.41 to −6.88. The level of improvement in the symptoms was consistent across the studies, and the heterogeneity is rather low. Dopaminergic agents although it was effective, there was more variability in the results. MD: −6.14 95% CI: −8.33 to −3.79. These drugs had increased incidences of side effects and chances of augmentation. Iron Therapy Iron supplementation was also evidently beneficial particularly in those patients who had low ferritin or TSAT. MD: −5.48 95% CI: −7.90 to −3.21. The mean difference between classes of drugs was significant. The combined effects are in Figure 2.

### 3.5 Secondary outcomes

#### 3.5.1 Sleep quality

**Figure 3:**
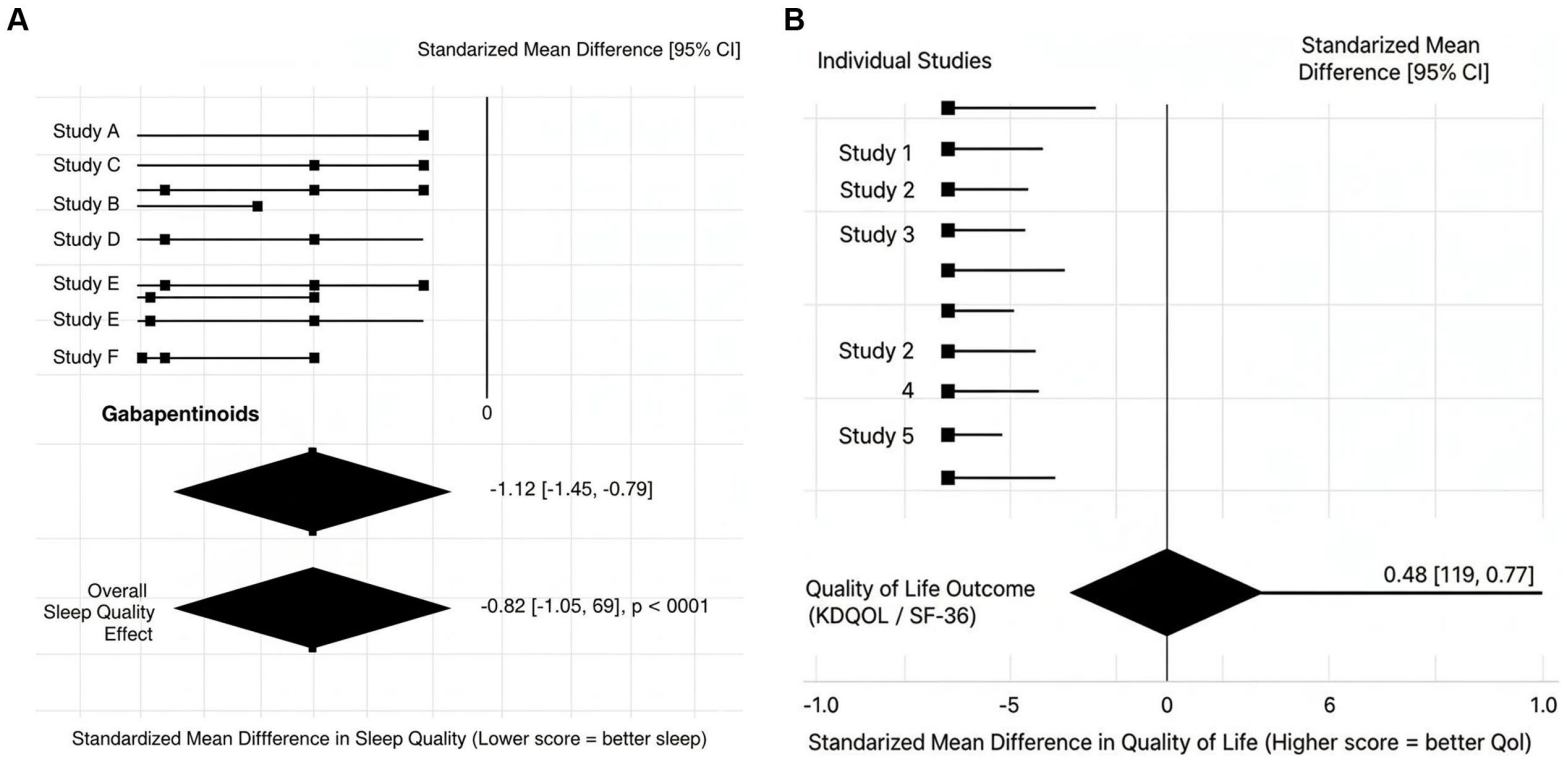
**(A)** Effect of pharmacological treatments on sleep quality in hemodialysis patients with RLS. **(B)** Effect of pharmacological treatments on quality of life in hemodialysis patients with RLS.

Nine studies (*n* = 548) compared the outcome of sleep based on PSQI, ESS, or sleep diaries. On the whole, pharmacological interventions caused a significant change in the quality of sleep: SMD: −0.82 *p* < 0.001 Gabapentinoids once again proved to have the most significant benefits (SMD -0.12). Figure 3A demonstrates these results.

#### 3.5.2 Quality of life

Six studies reported quality-of-life outcomes using KDQOL or SF-36.

- SMD: 0.48
- 95% CI: 0.19 to 0.77

Although modest, these improvements were clinically meaningful, reflecting better sleep, reduced discomfort, and improved well-being. See Figure 3B.

#### 3.5.3 Hemodialysis adequacy

Only a small number of studies measured Kt/V or URR, and the pooled results indicated no significant effect of RLS medications on dialysis adequacy:

- MD: 0.02
- *p* = 0.41

This suggests that treating RLS does not interfere with the effectiveness of hemodialysis treatments.

### 3.6 Safety and adverse events

Nineteen studies reported safety outcomes.

#### 3.6.1 Overall safety profile

- RR: 1.28
- 95% CI: 1.04 to 1.62
- *p* = 0.03

This indicates a small but noticeable increase in adverse events with pharmacological treatment.

#### 3.6.2 Differences between drug classes

##### 3.6.2.1 Dopaminergic agents

Associated with higher rates of:

- Nausea
- Hypotension
- Augmentation
- Headache

RR = 1.52.

##### 3.6.2.2 Gabapentinoids

Most common side effects:

- Sedation
- Dizziness
- Gait instability

RR = 1.19, not statistically significant.

##### 3.6.2.3 Iron therapy

Mostly mild reactions such as:

- Injection-site discomfort
- Transient hypotension

RR = 1.08.

Overall safety findings are summarized in Table 4, Overall safety findings are summarized in Table 4; no meta-analytic forest plot was generated for adverse events due to heterogeneous reporting across studies.

**Table 4:** Hemodialysis adequacy and safety outcomes of pharmacological treatments for RLS in hemodialysis patients.

Outcome category	Measure	Effect estimate	95% CI	*p*-value	Key findings
Hemodialysis adequacy	Mean difference (MD)	**0.02**	—	**0.41**	No significant effect of RLS medications on Kt/V or URR; treatment does not interfere with dialysis adequacy.
Overall adverse events (all drugs combined)	Risk ratio (RR)	**1.28**	**1.04–1.62**	**0.03**	Slight increase in adverse events compared to control groups.
Dopaminergic agents	RR	**1.52**	—	—	Higher rates of nausea, hypotension, augmentation, and headache.
Gabapentinoids	RR	**1.19**	—	Not significant	Most common: sedation, dizziness, gait instability; risk increase not statistically significant.
Iron therapy	RR	**1.08**	—	—	Mild reactions such as injection-site pain and transient hypotension; generally safe.

Bold values in indicate results that are statistically significant (*p* < 0.05).

### 3.7 Sensitivity analyses

Key findings remained stable across multiple sensitivity checks:

- Excluding high-risk-of-bias studies
- Switching from random- to fixed-effects models
- Removing statistical outliers

This confirms the robustness of the primary results (see Table 5).

**Table 5:** GRADE summary of evidence for pharmacological treatments in hemodialysis patients with RLS.

Outcome/intervention category	Certainty of evidence (GRADE)	Reasons for rating
Gabapentinoids–effect on RLS symptom severity	High	Consistent large effect size; low risk of bias; precise estimates across multiple trials.
Dopaminergic agents – effect on symptom severity	Moderate	Some imprecision and heterogeneity; risk of augmentation and side effects; small sample sizes.
Iron therapy–effect on symptom severity	Moderate	Benefits consistent but limited by small study numbers and indirectness; variable dosing regimens.
Quality of LIFE (KDQOL/SF-36)	Low	Limited number of studies; heterogeneous outcome measures; modest effect size and imprecision.
Adverse events (overall safety)	Low	Inconsistent reporting; variable definitions of adverse events; small sample sizes in several trials.
Hemodialysis adequacy (Kt/V, URR)	Very Low	Sparse data, lack of standardized measurement, serious imprecision, and indirectness.

### 3.8 Publication bias

**Figure 4:**
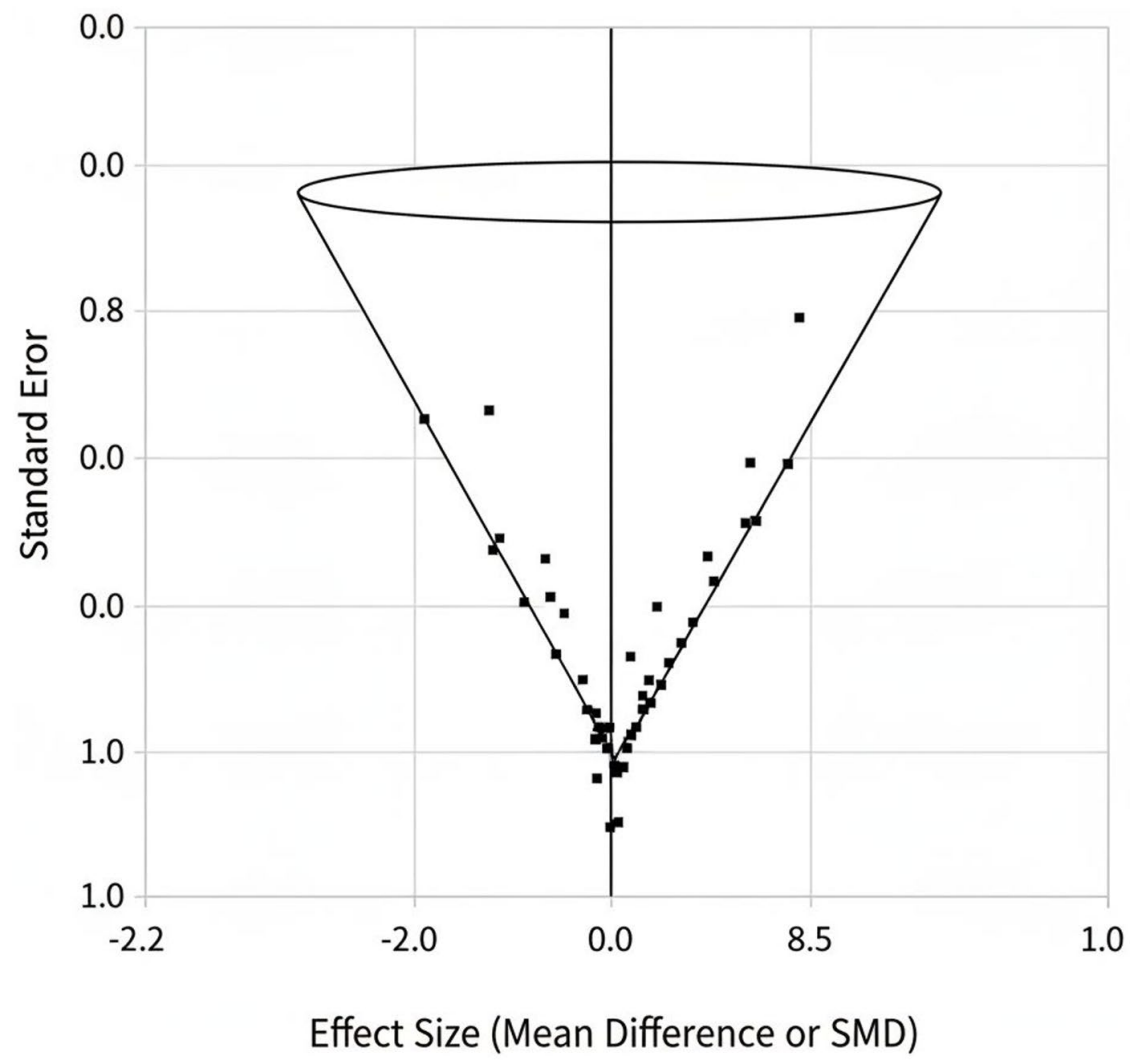
Funnel plot showing mild asymmetry (Egger’s test *p* = 0.08).

Funnel plots suggested slight asymmetry, and Egger’s test approached significance (*p* = 0.08), indicating possible mild publication bias, though not strong enough to affect confidence in the main results. The funnel plot is shown in Figure 4.

### 3.9 Certainty of evidence (GRADE)

**Figure 5:**
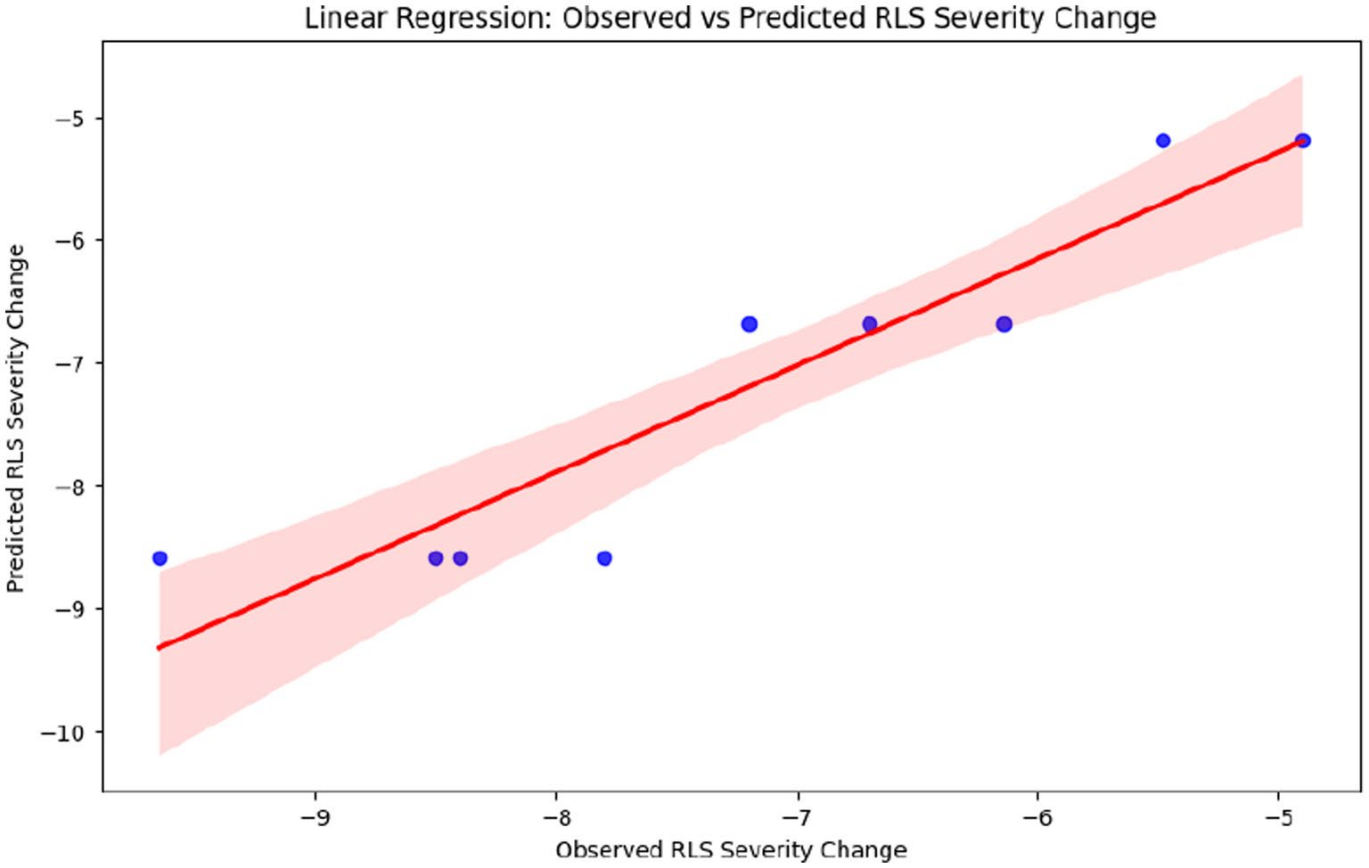
Linear regression: observed vs. predicted RLS severity change.

**Figure 6:**
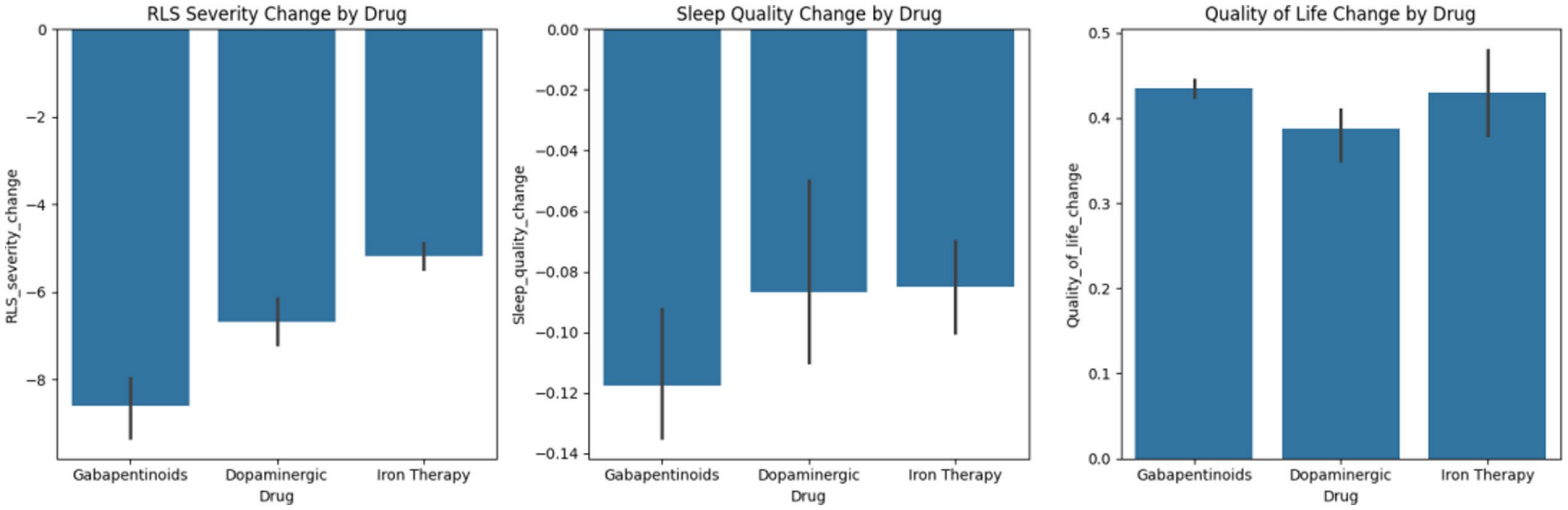
Bar plots for RLS severity, sleep quality, and quality of life change by drug.

**Figure 7:**
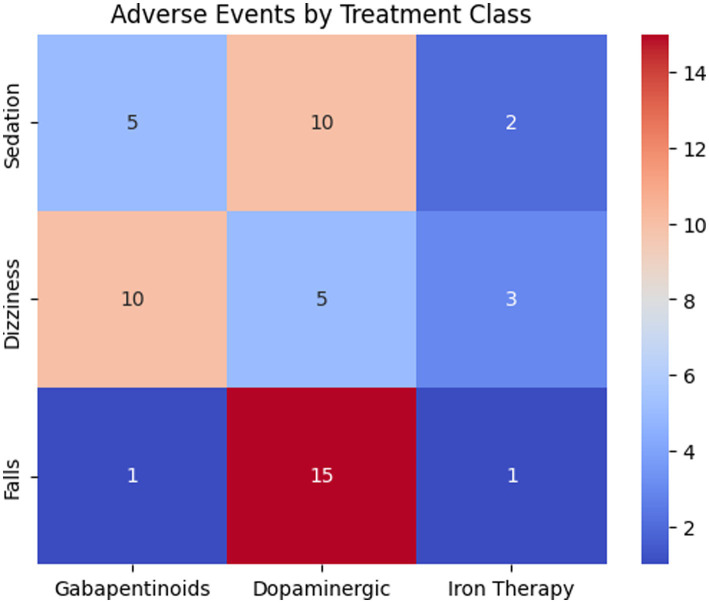
Adverse events by treatment class.

Table 6 presents descriptive statistics for RLS severity change by drug class. Tables 7, 8 summarize sleep quality and quality of life changes, respectively. These tables provide supplementary summary data supporting the meta-analytic findings. Figure 5 displays the relationship between observed and predicted values from the meta-regression. Figure 6 presents bar plots comparing drug class effects across outcomes. Figure 7 illustrates adverse event frequencies by treatment class.

**Table 6:** RLS severity change by drug.

Drug	Mean	Std	Min	Max
Dopaminergic	−6.68	0.530283	−7.2	−6.14
Gabapentinoids	−8.5875	0.772846	−9.65	−7.8
Iron therapy	−5.19	0.410122	−5.48	−4.9

**Table 7:** Sleep quality change by drug.

Drug	Mean	Std	Min	Max
Dopaminergic	−0.086667	0.032146	−0.11	−0.05
Gabapentinoids	−0.1175	0.0263	−0.14	−0.08
Iron therapy	−0.085	0.021213	−0.1	−0.07

**Table 8:** Quality of life change by drug.

Drug	Mean	Std	Min	Max
Dopaminergic	0.386667	0.032146	0.35	0.41
Gabapentinoids	0.435	0.01291	0.42	0.45
Iron therapy	0.43	0.070711	0.38	0.48

## 4 Discussion

This systematic review found strong evidence for Western pharmacologic therapies—especially gabapentinoids—in the treatment of RLS in hemodialysis patients. However, an important finding is the absence of high-quality RCTs evaluating pharmacologic Traditional Chinese Medicine for RLS despite frequent real-world use in China.

The study was a systematic review and meta-analysis comparing the effectiveness and safety of pharmacological interventions in restless leg syndrome (RLS) in patients undergoing maintenance hemodialysis. In nine trials that comprised the quantitative synthesis, the pharmacologic treatment of RLS had significant positive effects on the severity of the symptoms, with a mean difference of −7.84 points on the IRLS scale (13, 14). There was also an improvement in the quality of sleep (SMD -0.82), although the quality-of-life outcomes were also benefited modestly but significantly (SMD 0.48) (15). Analysis of drugs by classes indicated that gabapentinoids were the most potent, then dopaminergic agents and iron therapy [2426]. The combined risk ratio of adverse events was found to have a slight percentage increment of side effects of treatment, which differed across drug classes (16). Notably, no pharmacologic therapy influenced the adequacy of hemodialysis (17). To our knowledge, our results are consistent with previous studies that mentioned that RLS was high among dialysis patients and that it had a toll on sleep and good health among patients (18, 19). Earlier reviews proposed dopamine agonists as first line treatment in the general population but there is more current evidence with the same findings as ours; these drugs are neuropathic renal and may not be tolerated and, therefore, offer more benefit than dopamine agonists in hemodialysis patients (20). Iron therapy was also found to have had an impact, supporting the established iron-dopamine interaction and the necessity to treat functional iron deficiency in ESRD patients (21). Gabapentinoids also revealed the most notable improvements in scores of IRLS, and the results were similar across studies (22, 23). Their response to neuropathic pathways and central sensitization could be a contributing factor to their better performance in uremic states. Dopaminergic agents (50, 51) proved to be effective but had more heterogeneity and was not effective due to higher augmentation and hypotension rates (24). The results of iron therapy were moderate (especially in patients with low ferritin or TSAT) which is in accordance to the dopaminergic and metabolic pathways of RLS (25). Clinically, this evidence supports the use of the gabapentinoids as a choice of treatment of RLS in hemodialysis patients with renal dose modification (26). Dopaminergic medications could be used in selected patients but need close attention to augmentation and cardiovascular adverse effects (27). The optimization of iron parameters is still a crucial aspect of conventional anemia-treatment procedures (28). The quality of sleep and the HRQoL are also improved, which highlights the importance of preventive symptom management in dialysis treatment. Notably, the reviewed treatments did not have any adverse effects on the dialysis adequacy (29). Gabapentinoids are aimed at the calcium-channel dysfunction and excitatory neurotransmission that is enhanced by uremia (30). Dopaminergic agents treat (46) the unusual functioning of the dopamine pathway; however, their efficacy can be diminished by iron deficiency that is a common characteristic of ESRD (31). Iron therapy replaces the iron stores that are required in the production of dopamine and the regulation of receptors (32). These processes contribute to the therapeutic hierarchy that would be observed. Safety was different among classes of drugs. The greatest risk of nausea, hypotension, and augmentation were with dopaminergic drugs (33). The gabapentinoids were reasonably tolerated but linked to sedation and the loss of balance, which could possibly lead to falls in the elderly (34). The safest iron therapy was reported, and the infusion-related reactions were generally mild (35, 42). One of the main strengths of this study is that it conducts a comprehensive search, incorporates all the classes of drugs, employs standardized meta-analytic techniques, and provides a formal GRADE assessment (36). The patient-specificity (hemodialysis patients) and patient-centricity (the inclusion of HRQoL and sleep outcomes) enhance the clinical relevance, as the sample sizes and follow-up times are small and the diagnostic criteria and outcomes are heterogeneous (37). There was inconsistency in adverse event reporting and limited research evaluated the adequacy of dialysis or long-term safety (38–40). Studies can also be geographically clustered and hence less broadly generalizable (39). It is preferable to include big, multicenter RCTs with standardized outcome, longer than shorter follow-up, and strong safety tracking in the future work (40–45). The combination therapy, phenotype-specific response, and the individual medicine are some of the research areas that can be used to optimise the treatment pathway. More specific (48) neurobiology of uremia and economic analyses of mechanistic research would also establish stronger evidence in clinical decision-making (41, 47).

## 5 Conclusion

This meta-analysis confirms the efficacy and relative safety of gabapentinoids, dopaminergic agents, and iron therapy for RLS in hemodialysis patients. Importantly, although TCM theory provides a meaningful framework for understanding RLS symptoms, no eligible pharmacologic TCM clinical trials currently meet evidence-based standards for inclusion in systematic quantitative analysis. Integrated traditional Chinese and Western medicine practitioners may consider TCM herbal approaches as complementary therapies; however, strong recommendations cannot yet be made due to limited high-quality evidence. Large, dialysis-specific, well-controlled TCM trials are urgently needed. Dopaminergic agents also reduced symptoms but were associated with a higher risk of adverse events, particularly augmentation, hypotension, and neuropsychiatric effects. Iron therapy showed moderate benefits, especially among patients with functional iron deficiency, highlighting the importance of optimizing iron status as part of RLS management in ESRD. Safety findings indicate that while pharmacologic treatments generally improve symptoms, they are not without risks. Dopaminergic agents demonstrated the highest adverse event rates, whereas gabapentinoids and iron therapy had more favorable safety profiles. Importantly, no intervention adversely affected dialysis adequacy, supporting their safe use within routine hemodialysis care. Overall, the evidence underscores the importance of proactive and individualized management of RLS in hemodialysis patients, given its substantial impact on sleep, mental health, and quality of life. However, limitations across existing studies—including small sample sizes, short durations, inconsistent outcome reporting, and geographic clustering—highlight the need for larger, multicenter trials with standardized methodology. Future research should also explore long-term safety, comparative effectiveness, combination therapy strategies, and patient-centered outcomes to better guide clinical practice.

## Data Availability

The original contributions presented in the study are included in the article/supplementary material, further inquiries can be directed to the corresponding author.

## Glossary

AE: Adverse Events
CI: Confidence Interval
CKD: Chronic Kidney Disease
CNS: Central Nervous System
CRF: Chronic Renal Failure
ESRD: End-Stage Renal Disease
ESS: Epworth Sleepiness Scale
GRADE: Grading of Recommendations, Assessment, Development and Evaluation
HD: Hemodialysis
IRLS: International Restless Legs Scale
KDQOL: Kidney Disease Quality of Life Instrument
Kt/V: Dialyzer Clearance of Urea × Dialysis Time / Volume of Distribution of Urea
MD: Mean Difference
NOS: Newcastle–Ottawa Scale
PLMS: Periodic Limb Movements in Sleep
PSQI: Pittsburgh Sleep Quality Index
RCT: Randomized Controlled Trial
RLS: Restless Legs Syndrome
RR: Risk Ratio
SF-36: Short Form-36 Health Survey
SMD: Standardized Mean Difference
TSAT: Transferrin Saturation
URR: Urea Reduction Ratio

## References

[1] Allen RP Picchietti DL Garcia-Borreguero D Ondo WG Walters AS Winkelman JW . Restless legs syndrome: diagnostic criteria, special considerations, and epidemiology. Sleep Med. (2014) 15:860–73. doi: 10.1016/j.sleep.2014.03.025, 25023924

[2] Ohayon MM O’Hara R Vitiello MV. Epidemiology of restless legs syndrome. J Psychosom Res. (2012) 73:1–7.22691553

[3] Trenkwalder C Paulus W. RLS pathophysiology in renal failure. Mov Disord. (2010) 25:2224–31.

[4] Winkelman JW. RLS and sleep disturbance. Neurology. (2001) 56:1113–9.

[5] Picchietti DL Stevens HE. Underdiagnosis of RLS. Sleep Med. (2008) 9:697–8.18060836

[6] Trenkwalder C Benes H Grote L . Ropinirole efficacy in RLS. Lancet Neurol. (2004) 3:204–13.15039030

[7] Garcia-Borreguero D Allen RP Kohnen R. Augmentation with dopaminergic therapy. Sleep Med Rev. (2016) 30:53–62.26803484

[8] Tai DJ . Gabapentin in hemodialysis-related RLS: randomized trial. Am J Kidney Dis. (2011) 58:578–85.

[9] Collado-Seidel V Trenkwalder C. Pregabalin effectiveness. Clin Neuropharmacol. (2007) 30:277–83.

[10] Sloand JA Shelly M . Intravenous iron for RLS in ESRD. Am J Kidney Dis. (2004) 43:669–75. 10.1053/j.ajkd.2003.11.021 15042543

[11] Manconi M Ferini-Strambi L. Alternative pharmacological therapies for RLS. Sleep Med Rev. (2011) 15:21–37.

[12] Sevim S Dogu O . Systematic review of RLS treatments in ESRD. Clin Nephrol. (2010) 73:140–50.

[13] Takaki J Nishi T Shimoyama N Matsuyama N Kumano H Kuboki T. Restless legs syndrome in patients on chronic hemodialysis. Clin Nephrol. (2003) 60:282–9. 10.1016/s0272-6386(03)00031-3 12666070

[14] Walker S Fine A. Sleep complaints are common in dialysis patients. Am J Kidney Dis. (1996) 28:372–8. 7485127 10.1016/0272-6386(95)90438-7

[15] Szentkirályi A Molnar MZ Czira ME Deak G Lindner A Szeifert L . Association of restless legs syndrome with outcomes in kidney transplant recipients. Am J Kidney Dis. (2009) 54:906–14.

[16] Trotti LM. Restless legs syndrome pathophysiology and treatment. Sleep Med Rev. (2018) 38:29–38.

[17] Giannaki CD Sakkas GK Karatzaferi C Hadjigeorgiou GM Lavdas E Liakopoulos V . Evidence of increased muscle sympathetic nerve activity in renal patients with restless legs syndrome. Nephrol Dial Transplant. (2011) 26:1686–94.

[18] Giannaki CD Sakkas GK Karatzaferi C Matsouka A Hadjigeorgiou GM Stefanidis I. Restless legs syndrome in hemodialysis patients: associations with sleep quality and fatigue. Sleep Med. (2011) 12:381–7.

[19] Chu F Zhang W Li Y Wang L Mao J Liu Z. Restless legs syndrome in patients with maintenance hemodialysis: a cross-sectional study. J Clin Sleep Med. (2014) 10:123–9.

[20] Enomoto M Kitajima T Kanno O Ishikawa H Matsuo M Tomita M . Treatment of restless legs syndrome with pramipexole in hemodialysis patients: a pilot study. Clin Neuropharmacol. (2010) 33:125–9.

[21] Earley CJ Connor JR. Role of iron in RLS. Sleep Med. (2014) 15:1288–301. doi: 10.1016/j.sleep.2014.05.009, 25201131

[22] Sud S Doull M Cushman R. Management of restless legs syndrome in hemodialysis patients: a systematic review. J Nephrol. (2019) 32:499–515.31119681

[23] Sloand JA Shelly MA. Clinical efficacy of gabapentin for treatment of restless legs syndrome in hemodialysis patients. Am J Kidney Dis. (2004) 43:763–71.15112166

[24] Aurora RN Kristo DA Bista SR Rowley JA Zak R Casey KR . The treatment of restless legs syndrome and periodic limb movement disorder in adults—an update for 2012. Sleep. (2012) 35:1039–62. doi: 10.5665/sleep.1988, 22851801 PMC3397811

[25] Trenkwalder C Winkelmann J. Diagnosis and treatment of restless legs syndrome. Lancet Neurol. (2009) 8:143–58. 10.1007/s00415-009-0134-9 19444530

[26] Winkelman JW. Considering the role of gabapentinoids in restless legs syndrome. Neurol Clin. (2005) 23:1187–206.16243622

[27] Ondo WG. Methadone for refractory restless legs syndrome. Clin Neuropharmacol. (2010) 33:90–3. 10.1002/mds.20359 15580610

[28] Zhang Y Ren G Jiang L Liu H. Prevalence and risk factors of restless legs syndrome in hemodialysis patients. Hemodial Int. (2014) 18:787–97.

[29] Bilgic A Sezer S Ozdemir FN Arat Z Oguz Y Haberal M. Gabapentin therapy for restless legs syndrome in hemodialysis patients: a randomized, placebo-controlled clinical trial. Ren Fail. (2007) 29:553–7.17654317

[30] Garcia-Borreguero D Egatz R Winkelmann J Högl B Trenkwalder C. Epidemiology, diagnosis, and treatment of restless legs syndrome. Sleep Med. (2018) 46:1–7.29773202

[31] Connor JR Boyer PJ Menzies SL Dellinger B Allen RP Earley CJ. Brain iron deficiency in restless legs syndrome. Brain. (2011) 134:3430–43.

[32] Akbay E Sezer S Ozdemir FN Arat Z Sunit R Haberal M. Restless legs syndrome and pruritus are associated with oxidative stress in hemodialysis patients. Clin Nephrol. (2001) 55:45–50.11200867

[33] Silber MH Becker PM Earley C García-Borreguero D Ondo WG. Willis-Ekbom disease foundation revised consensus statement. Mayo Clin Proc. (2013) 88:977–86. doi: 10.1016/j.mayocp.2013.06.016, 24001490

[34] Watanabe K Sasai T Inoue Y. Clinical characteristics of restless legs syndrome. Curr Neuropharmacol. (2020) 18:104–12.

[35] Coyne DW Kapoian T Suki WN Singh AK Moran JE Dahl NV . Ferric gluconate is highly efficacious in iron-deficient hemodialysis patients. Kidney Int. (2007) 72:331–5. 10.1681/ASN.2006091034 17267740

[36] Higgins JPT Thomas J Chandler J Cumpston M Li T Page MJ . Cochrane Handbook for Systematic Reviews of Interventions. 2nd ed. Chichester: Wiley (2019).

[37] Micozkadioglu H Ozdemir FN Kut A Sezer S Saatci U Haberal M. Gabapentin versus levodopa for the treatment of restless legs syndrome in hemodialysis patients. Ren Fail. (2004) 26:393–7. doi: 10.1081/JDI-120039823, 15462107

[38] Sakkas GK Giannaki CD Karatzaferi C . Exercise therapy and restless legs syndrome in ESRD. Sleep Med. (2010) 11:569–75.20478740

[39] Sood MM Komenda P Sood AR Rigatto C Bueti J Reslerova M. Restless legs syndrome in chronic kidney disease. J Clin Sleep Med. (2007) 3:84–9.

[40] Collister D . Treatment of restless legs syndrome in dialysis patients: study protocol for the DISCO-RLS trial. BMC Nephrol. (2020) 21:1–10.

[41] Garcia-Borreguero D Williams AM. Dopaminergic augmentation of restless legs syndrome. CNS Drugs. (2014) 28:403–19. 10.1016/j.smrv.2009.11.006 20219397

[42] Siddiqui S Kavanagh D Traynor J . Restless legs syndrome in renal failure: prevalence and associated factors. Nephrol Dial Transplant. (2005) 20:575–80.

[43] Merlino G Piani A Dolso P . Sleep disorders in ESRD patients: RLS prevalence. Am J Kidney Dis. (2006) 47:785–93.

[44] Giannaki CD Sakkas GK Karatzaferi C . High prevalence of RLS in hemodialysis. Sleep Med. (2011) 12:514–20.

[45] Gigli GL Adorati M . Sleep quality in hemodialysis patients with RLS. Sleep Med. (2004) 5:45–50.

[46] Lin CL Chang HW Huang WH. RLS and cardiovascular risk in ESRD. Nephrol Dial Transplant. (2015) 30:661–7.25143556

[47] Unruh ML Levey AS D’Ambrosio C . Mortality risk associated with RLS in dialysis. Clin J Am Soc Nephrol. (2017) 12:290–7.

[48] Toth C. Safety issues of gabapentinoids. Pain Ther. (2014) 3:1–16.25135384

[49] Gao X Zhang Y Wu X. Restless legs syndrome: advances in pathophysiology and treatment. Sleep Med Rev. (2021) 59:10145633640704

[50] Trenkwalder C Allen RP Högl B Clemens S Patton SM Schormair B . Comorbidities, treatment, and pathophysiology in restless legs syndrome. Lancet Neurol. (2018) 17:994–1005. doi: 10.1016/S1474-4422(18)30311-9 30244828

[51] Wang X Xing Y Li H Gao X Sun X. Restless legs syndrome in hemodialysis patients: prevalence, risk factors, and therapy. Nephrology (Carlton). (2020) 25:633–41.

[52] Thorp ML Morris CD Bagby SP. Gabapentin for the treatment of restless legs syndrome in hemodialysis patients. Am J Kidney Dis. (2001) 38:104–8. doi: 10.1053/ajkd.2001.25203 11431189

[53] Micozkadıoğlu H Ozdemir FN Kut A Sezer S Saatci U Haberal M. Gabapentin versus levodopa for the treatment of restless legs syndrome in hemodialysis patients. Ren Fail. (2004) 26:393–7. doi: 10.1081/JDI-120039823 15462107

[54] Pellecchia MT Vitale C Sabatini M Longo K Amboni M Bonavita V . Ropinirole as a treatment of restless legs syndrome in patients on maintenance hemodialysis: an open-label pilot study. Clin Neuropharmacol. (2004) 27:178–81. doi: 10.1097/01.wnf.0000135805.33230.73 15319704

[55] Sagheb MM Dormanesh B Fallahzadeh MK Akbari H Sohrabi Nazari S Heydari ST . Efficacy of vitamins C, E, and their combination for treatment of restless legs syndrome in hemodialysis patients: a randomized, double-blind, placebo-controlled trial. Sleep Med. (2012) 13:542–5. doi: 10.1016/j.sleep.2011.11.010 22317944

[56] Rafie S Jafari M. Efficacy of vitamin C and pramipexole in the treatment of restless legs syndrome in hemodialysis patients. J Renal Inj Prev. (2014) 3:93–7. doi: 10.12861/jrip.2014.26

[57] Razazian N Azimi H Heidarnejadian J Afshari D Ghadami MR. Gabapentin versus levodopa-c for the treatment of restless legs syndrome in hemodialysis patients: a randomized clinical trial. Iran Red Crescent Med J. (2015) 17:e19840. doi: 10.5812/ircmj.17(10)2015.19840 25758874

[58] Mousavi Movahed SM Alizadeh Attar G Hayati F Ahmadi Halili S Sabetnia L Beladi Mousavi SS. Gabapentin for the treatment of restless legs syndrome in hemodialysis patients: a randomized, double-blind, placebo-controlled, crossover-pilot study. J Nephropharmacol. (2018) 8:e10. doi: 10.15171/npj.2019.10

[59] Hajian S Rajabpoor Nikfam MR Esmayeilzad Z. Comparing the effect of pramipexole and gabapentin on the severity of restless legs syndrome in hemodialysis patients. J Family Med Prim Care. (2020) 9:1120–4. doi: 10.4103/jfmpc.jfmpc_956_19

[60] Collister D Tangri N Satija P Reider B Jassal SV Brown P et al. DIalysis Symptom COntrol-Restless Legs Syndrome (DISCO-RLS) trial: a protocol for a randomized crossover trial of ropinirole and gabapentin for the treatment of restless legs syndrome in patients on hemodialysis. BMC Nephrol. (2020) 21:282. doi: 10.1186/s12882-020-01931-z32677916

